# Progressive alterations of left atrial and ventricular volume and strain across chronic kidney disease stages: a speckle tracking echocardiography study

**DOI:** 10.3389/fcvm.2023.1197427

**Published:** 2023-09-06

**Authors:** Hoai Thi Thu Nguyen, Chien Van Do, Dieu Thi Vu Dang, Loi Doan Do, Linh Huu Doan, Ha Thi Viet Dang

**Affiliations:** ^1^ Vietnam National Heart Institute, Bach Mai Hospital, Hanoi, Vietnam; ^2^ VNU University of Medicine and Pharmacy, Vietnam National University, Hanoi, Vietnam; ^3^ Department of Cardiovascular Intensive Care Unit, 108 Military Central Hospital, Hanoi, Vietnam; ^4^ Department of Cardiology, Hanoi Medical University, Hanoi, Vietnam; ^5^ Department of Internal Medicine, Hanoi Medical University, Hanoi, Vietnam; ^6^ Center of Nephrology, Urology and Dialysis, Bach Mai Hospital, Hanoi, Vietnam

**Keywords:** chronic kidney disease, speckle tracking echocardiography, left atrial strain, left ventricular strain, cardiovascular disease

## Abstract

**Background:**

It has been a scarcity of evidence regarding differences in left ventricular (LV) and left atrial (LA) size and strain changes across stages of chronic kidney disease (CKD) and which echocardiographic parameters could be utilized to predict the decline of glomerular filtration rate (GFR).

**Objectives:**

This study aimed to evaluate the alterations of LV and LA strain across the reduction of renal function and potential echocardiographic parameters which could be correlated with the GFR decline among patients with CKD.

**Method:**

A cross-sectional study was conducted on 169 CKD patients at Bach Mai General Hospital, Hanoi, Vietnam from April to November 2022. Demographic, clinical and laboratory characteristics of patients were collected. Transthoracic echocardiography was performed to measure LV and LA size and strains. Jonckheere-Terpstra test was used to measure the tendency of change. Multivariate linear regression models were performed to find associations between different echocardiographic parameters and renal function reduction.

**Results:**

The number of patients with CKD stages 1, 2, 3, 4, and 5 was 21 (12.4%), 28 (16.6%), 27 (16.0%), 22 (13.0%) and 71 (42.0%), respectively. CKD severity was positively associated with LV diastolic and systolic diameters, LV mass, E/e' ratio, and maximal tricuspid regurgitation velocity (TR max), and negatively correlated with the LV global longitudinal strain. Higher severity of CKD stage was associated with higher LA diameter, LA strain, and volume in four and two-chamber views, and lower LA reservoir and conduit function. Left ventricular mass (*β* = 0.068), ejection fraction (*β* = 0.112) and left atrial reservoir (*β* = −0.077) were associated with reduced GFR.

**Conclusion:**

Left ventricular mass, ejection fraction, and atrial longitudinal strain by STE should be done at the earlier stages of CKD patients for better follow-up of GFR decline.

## 1. Introduction

Chronic kidney disease (CKD) significantly contributes to the global burden of diseases given the matter that it affected around 10% of the global population (or approximately 800 million people) (1). In literature, CKD was found to be associated with cardiovascular diseases (CVD), particularly, the estimated glomerular filtration rate (eGFR) <60 ml/min/m^2^ was associated with a higher risk of cardiovascular morbidity and mortality after adjusting to other independent factors (2). Prompt diagnosis and treatment can prevent the development of CKD to end-stage renal disease (ESRD) (3). The classic signs of cardiomyopathy in CKD are altered left ventricular (LV) mass and function. In high-risk individuals with conditions like hypertension or heart failure, the left atrium, a heart chamber that is incredibly sensitive to fluid overload and diastolic dysfunction, is a stand-alone predictor of mortality and unfavorable cardiovascular (CV) events (4).

Recently, a new class of cardiomyopathy known as uremic in CKD can be identified by imaging modalities such as echocardiography and cardiac magnetic resonance (CMR). This cardiomyopathy has distinctive phenotypes with important changes such as left ventricular (LV) enlargement, left atrial (LA) dilatation, diastolic dysfunction, and decreased myocardial deformation which could indicate myocardial fibrosis (5–7). In literature, among ESRD children undergoing hemodialysis (HD), significant abnormalities in speckle tracking analysis of systolic and diastolic LV and LA phasic function were observed, and global longitudinal strain (GLS) was found to be associated with a change in blood pressure, and this novel non-invasive indicator could be used to measure the long-term impact on the risk of cardiovascular abnormalities among children (8). However, it has been a scarcity of evidence regarding differences in LV and LA size and strain changes across stages of CKD, and which echocardiographic parameters could be utilized to predict the decline of GFR. Therefore, this study aimed to evaluate the alterations of LV and LA strain across the reduction of renal function and potential echocardiographic parameters which could be correlated with the GFR decline among patients with CKD.

## 2. Materials and methods

### 2.1. Study population

A cross-sectional study was conducted at Bach Mai General Hospital, Hanoi, Vietnam from April to November 2022. All patients who were diagnosed with CKD according to KDIGO 2012 criteria (9), and visited the hospital for cardiovascular consultation were consecutively recruited in the study. The CKD stages were defined according to GFR values (9): Stage 1 (Normal—GFR > 90 ml/min), Stage 2 (Mild CKD—GFR = 60–89 ml/min), Stage 3 (Moderate CKD—GFR = 30–59 ml/min), Stage 4 (Severe CKD—GFR = 15–29 ml/min) and Stage 5 (End stage CKD—GFR < 15 ml/min). Patients who (1) disagreed to participate in the study; (2) had significant valvular diseases; (3) had surgical valve repair or replacement, (4) had global left ventricular ejection fraction (LVEF) <50%, (5) had arrhythmias (atrial fibrillation, flutter, or pacemaker implantation), (6) heart rate >100 beats per minute, and (7) had chronic lung obstructive disease and acute renal failure were excluded. All eligible patients were asked to give signed consent forms and underwent meticulous clinical examinations before participating in the study. A total of 169 patients were included in the study. The study protocol was approved by the Institutional Review Board of Bach Mai hospital (Code CH2022/QĐBV).

### 2.2. Data collection and measurement

When performing transthoracic echocardiography (TTE), information about age, gender, blood pressure, heart rate, and body composition was collected. Laboratory testing results and clinical information were simultaneously obtained from a hospital's electronic database. TTE was done using a Vivid E95 ultrasound system (General electric Vingmed Ultrasound, Horten, Norway) and an M5S transducer with a frequency of 1.5–4.5 MHz. To avoid circadian effects, all ultrasound tests were carried out by qualified doctors in the late afternoon on the same day. Echocardiographic exams were performed following the scheduled hemodialysis session (10).

Based on the most recent recommendations of the European Association of Cardiovascular Imaging and the American Society of Echocardiography, cardiac chamber measurements were performed (11). The dimensions and volumes of the left atrium were determined using the methods outlined by the European Society of Cardiology for quantifying cardiac chambers (11). The measurement of LA volume was conducted through the application of the disk summation algorithm, which bears resemblance to the methodology employed in the measurement of LV volume. The tracing of the LA endocardial borders was imperative in both the apical four- and two-chamber views. The LA anteroposterior (AP) measurement in the parasternal long-axis view was evaluated utilizing 2D echocardiography. The LA area was measured using planimetry techniques within the apical four- and two-chamber echocardiographic views (11).

The linear internal dimension parameters of the LV and its walls were measured in the parasternal long-axis view at the level of the mitral valve leaflet tips. LV mass was calculated using Devereux's formula and was indexed to body surface area (BSA). The modified Simpson's approach was used to compute LV ejection fraction (LVEF). The left atrial anteroposterior diameter (LAd) was evaluated from the parasternal long-axis view. After manual adjustment, LA volume was automatically computed and indexed to BSA. Pulmonary artery systolic pressure (PAPs) was determined by using tricuspid regurgitant (TR) jet velocity, inferior vena cava diameter, and the percentage of collapsibility into the formula:

$$\mathrm{PAPs}\;(\mathrm{mmHg})=4(\mathrm{TRmax}{)}^{2}+\mathrm{Right}\;\mathrm{atrial}\;\mathrm{pressure}\;(\mathrm{RAP})$$

Doppler pulsed-wave imaging was used in the apical four-chamber view to measure the velocities of the mitral inflow. Tissue Doppler Imaging (TDI) was used to determine average mitral annular velocities at the septal and lateral walls. The peak systolic velocity of the mitral annulus (s' wave), the peak early diastolic velocity of the mitral annulus (*E* wave), the peak late diastolic velocity of the mitral annulus (*e'* wave), the ratio of the two (*E/e'* wave), and Isovolumic relaxation time (IVRT) were all measured. Determination of LV diastolic inflow using Doppler techniques was conducted on the apical four-chamber section by placing the sample volume at the tip level. *E* and *A* peak velocities along with their respective *E/A* ratio and *E*-wave deceleration time were computed. By using TDI, the early (*e*') diastolic velocities were evaluated at the septal and lateral insertion sites of the annulus of the mitral valve. The average value from the two measurements was then calculated (12).

Apical four-chamber and two-chamber pictures were recorded during breath hold for the speckle tracking analysis using a reliable TTE recording at a frame rate of 60–90 frames per second. In each view, three consecutive cardiac cycles were acquired for offline analysis using certain software (EchoPAC, version 204, GE Vingmed Ultrasound, Horten, Norway). Trained medical professionals measured the LV and LA speckle tracking parameters. GLS was evaluated following seven steps according to the guideline of Negishi et al. (13).

For the LV GLS and LA strain analyses, zero strain was established at the start of the *P* wave on the ECG (14). Indeed, we found no difference in the selection of *P* wave as the time reference for defining zero-baseline for both LV and LA strain curves in comparison with the use of ventricular end-diastole (15). LA phasic strains included reservoir phase (LASr), conduit phase (LAScd) and contractility (LASct).

**Figure 1:**
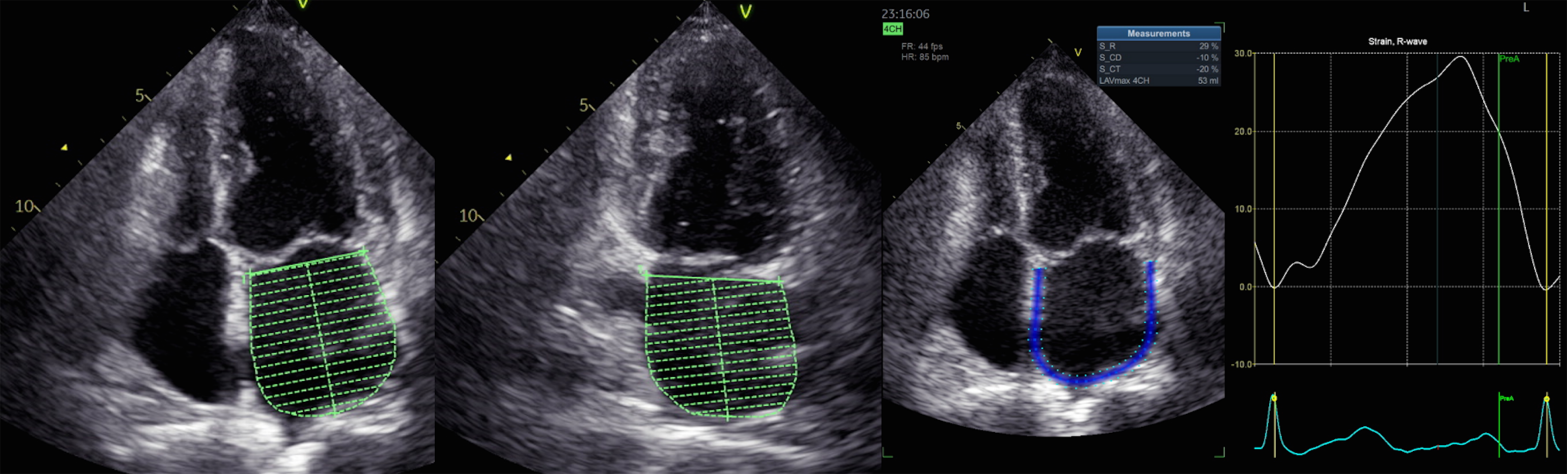
Measurement of left atrial volume (LAVI) and strain (LASr).

A specific echocardiographic image was elected for analysis (Figure 1). LA endocardial walls were manually traced in apical four- and two-chamber views using a point-and-click method. The software automatically identified the LA epicardial boundaries, generating a region of interest (ROI) that investigators might modify in terms of width and shape. In each view, LA walls were divided into six segments. Segments with poor image quality were excluded from the examination of the tracking of speckles. The evaluation of LA speckle tracking parameters (LA volume, E/e' ratio, LVEF, LV GLS) was conducted within the uniform apical four- and two-chamber image according to the previous guidelines (16, 17).

### 2.3. Reproducibility

The inter-observer and intra-observer variability pertaining to LA strain and strain rate were performed. Two separate occasions using a cohort of 10 patients selected randomly were conducted. One operator and two investigators, who were blinded to the prior outcomes, were involved in this study. In the context of the study, the calculated coefficients of variation for positive train and positive strain rate in the four-chamber view were found to be 10.2% (intra-observer) and 18.8% (inter-observer), and 9.4% (intra-observer) and 13.7% (inter-observer), respectively.

### 2.4. Statistical analysis

Statistical Package for Social Sciences (SPSS) version 25 was used to analyze the data. For categorical variables, descriptive statistics were presented as frequencies and percentages. Continuous variables were presented as mean (SD), or median (interquartile range-IQR). The Jonckheere-Terpstra test was applied to detect trends in continuous variables across the stages of CKD. The difference of categorical among the stages of CKD was evaluated using the Chi-square test. Multivariate linear regression was performed. No multicollinearity was found in the model. A *p*-value <0.05 was considered statistically significant.

## 3. Results

Among 169 patients, regarding clinical characteristics, Table 1 shows that there was no statistically significant difference in age, sex, or heart rate among the five CKD stages. Patients with higher CKD stages had a longer duration of disease (months) (*p* < 0.001), higher systolic blood pressure (mmHg) (*p* < 0.001) and higher diastolic blood pressure (mmHg) (*p* < 0.001) and different medication used (e.g., ACE-i/ARB, CCB, diuretics, SGLT2i, ARNI) (*p *< 0.001).

**Table 1:** Patients’ demographic and clinical characteristics.

	Stage 1 (*n* = 21)	Stage 2 (*n* = 28)	Stage 3 (*n* = 27)	Stage 4 (*n* = 22)	Stage 5 (*n* = 71)	*p*-value
Age, years, mean (SD)	42.1 (20.7)	48.6 (18.7)	58.8 (18.3)	55.3 (10.6)	49.6 (17.5)	0.783
Male, *n* (%)	9 (42.9%)	14 (50.0%)	12 (44.4%)	12 (54.6%)	40 (56.3%)	0.747
Duration of CKD, months, median (IQR)	12 (3–24)	6 (2–12)	12 (4–24)	22 (6–60)	36 (4–84)	**<0.001**
Heart rate, beats/min, mean (SD)	85 (11)	87 (12)	83 (10)	88 (24)	86 (9)	0.9634
Systolic BP, mmHg, mean (SD)	122.1 (16.9)	130.4 (22.1)	136.1 (21.0)	142.6 (28.2)	150.5 (22.0)	**<0.001**
Diastolic BP, mmHg, mean (SD)	80.1 (12.2)	79.3 (10.2)	81.9 (11.8)	85.2 (13.8)	87.5 (13.5)	**<0.001**
History related to CKD, *n* (%)						
Nephritis	10 (47.62%)	8 (28.57%)	2 (7.41%)	5 (22.73%)	6 (8.45%)	**<0.001**
Diabetes	3 (14.29%)	8 (28.57%)	5 (18.52%)	7 (31.82%)	12 (16.90%)	0.414
Hypertension	3 (14.29%)	12 (42.86%)	15 (55.56%)	13 (59.09%)	31 (71.83%)	**<0.001**
Others	2 (9.52%)	5 (17.86%)	6 (22.22%)	5 (22.73%)	12 (16.90%)	0.781
No history reported	6 (28.57%)	4 (14.29%)	4 (14.81%)	2 (9.09%)	8 (11.27%)	0.338
Medication						
ACE-i/ARB	15 (71.4%)	16 (57.1%)	18 (66.6%)	10 (45.4%)	5 (7.0%)	<0.001
CCB	3 (14.2%)	2 (7.1%)	6 (22.2%)	6 (27.2%)	45 (63.3%)	<0.001
SGLTi	12 (57.1%)	13 (46.4%)	17 (62.9%)	10 (45.4%)	21 (29.5%)	<0.001
ARNI	4 (19.0%)	5 (17.8)	8 (29.6%)	11 (50.0%)	12 (16.9%)	<0.001
Diuretics	11 (52.3%)	12 (46.4%)	15 (55.5%)	15 (68.1%)	57 (80.2%)	<0.001

Values are mean (SD), *n* (%), or median (interquartile range). The Jonckheere Terpstra test was used to assess for trend in continuous variables across the CKD stages. The chi-square test was used to assess the difference in categorical variables across the CKD stages. A *p* value <0.05 (bold) was considered to be statistically significant.

According to laboratory testing characteristics, Table 2 shows that there was a statistical association between more severe stages of CKD and lower GFR (*p* < 0.001), a higher concentration of serum urea (mmol/L) (*p* < 0.001) and creatinine (mmol/L) (*p* < 0.001). In terms of hematologic findings, lower hemoglobin (g/L) and RBC results (T/L) (*p* < 0.001) were related to more severe stages. Patients at the later stages had lower cholesterol levels (mmol/L), higher plasma albumin (g/L), higher plasma protein (g/L) and higher ferritin (ng/ml) (*p* < 0.05). No statistical difference in serum triglyceride, HDL-C, LDL-C, transferrin, and urine protein was found among CKD stages.

**Table 2:** Laboratory tests results characteristics.

	Stage 1 (*n* = 21)	Stage 2 (*n* = 28)	Stage 3 (*n* = 27)	Stage 4 (*n* = 22)	Stage 5 (*n* = 71)	*p*-value
Urea, mmol/L, Median (IQR)	5.4 (4.6–6.5)	6.2 (5.2–8.25)	8.7 (6.4–14)	15.85 (12–20)	23.8 (16.6–29.8)	**<0.001**
Creatinine, mmol/L, Mean (SD)	64.9 (12.1)	97.2 (16.3)	144.7 (32.1)	277.1 (58.7)	689.9 (261.2)	**<0.001**
GFR, ml/min/1.73 m^2^, mean (SD)	105.7 (15.2)	69.8 (7.3)	41.9 (8.2)	19.7 (5.0)	7.6 (3)	**<0.001**
RBC, T/L, mean (SD)	4.0 (0.9)	3.9 (0.8)	4.1 (0.6)	3.5 (0.8)	3.1 (0.6)	**<0.001**
Hemoglobin, g/L, mean (SD)	115.6 (23.4)	114.7 (23.1)	113.4 (23.4)	96.5 (18.6)	88.5 (17.6)	**<0.001**
Cholesterol, mmol/L, median (IQR)	5.1 (4.3–6.7)	5.2 (4.5–6.35)	5.1 (3.9–5.8)	4.55 (4.27–5.8)	4.6 (4–5.4)	**0.0298**
Triglyceride, mmol/L, median (IQR)	2.1 (1.6–3.0)	2.2 (1.8–3.35)	2.1 (1.8–2.8)	2.1 (1.66–2.8)	2.3 (2–2.8)	0.6151
HDL-C, mmol/L, median (IQR)	1.1 (0.9–1.3)	1 (0.9–1.3)	0.9 (0.9–1.1)	1 (0.9–1.1)	1 (0.9–1.1)	0.3869
LCL-C, mmol/L, median (IQR)	2.7 (2.3–3.6)	3.25 (2.4–3.85)	3.1 (2.8–3.2)	3.1 (2.8–3.5)	3.1 (2.6–3.4)	0.6938
Plasma albumin, g/L, mean (SD)	29.5 (9.8)	30.7 (8.6)	35.8 (5.5)	34.0 (7.4)	35.6 (5.6)	**0.0106**
Urine protein, g/L, median (IQR)	0.3 (0.15–10)	0.75 (0.3–3)	0.3 (0.15–1)	1.6 (1–3)	1 (0–3)	0.9304
Ferritin, ng/ml, median (IQR)	368 (330–510)	382.5 (300–496)	432 (340–638)	574.4 (428–851)	530.2 (304–907.9)	**0.0074**
Transferrin, ng/ml, median (IQR)	198 (160–207)	193 (162–210)	198 (170–210)	173.5 (147–190)	183 (160–200)	0.2612
Plasma protein, g/L, median (IQR)	62 (58–67)	65 (56–70)	66 (61–71)	65 (60–73.2)	68 (64–72.6)	**<0.001**

Values are mean (SD), *n* (%), or median (interquartile range). The Jonckheere Terpstra test was used to assess for trend in continuous variables across the CKD stages. The chi-square test was used to assess the difference in categorical variables across the CKD stages. A *p* value <0.05 (bold) was considered to be statistically significant.

**Figure 2:**
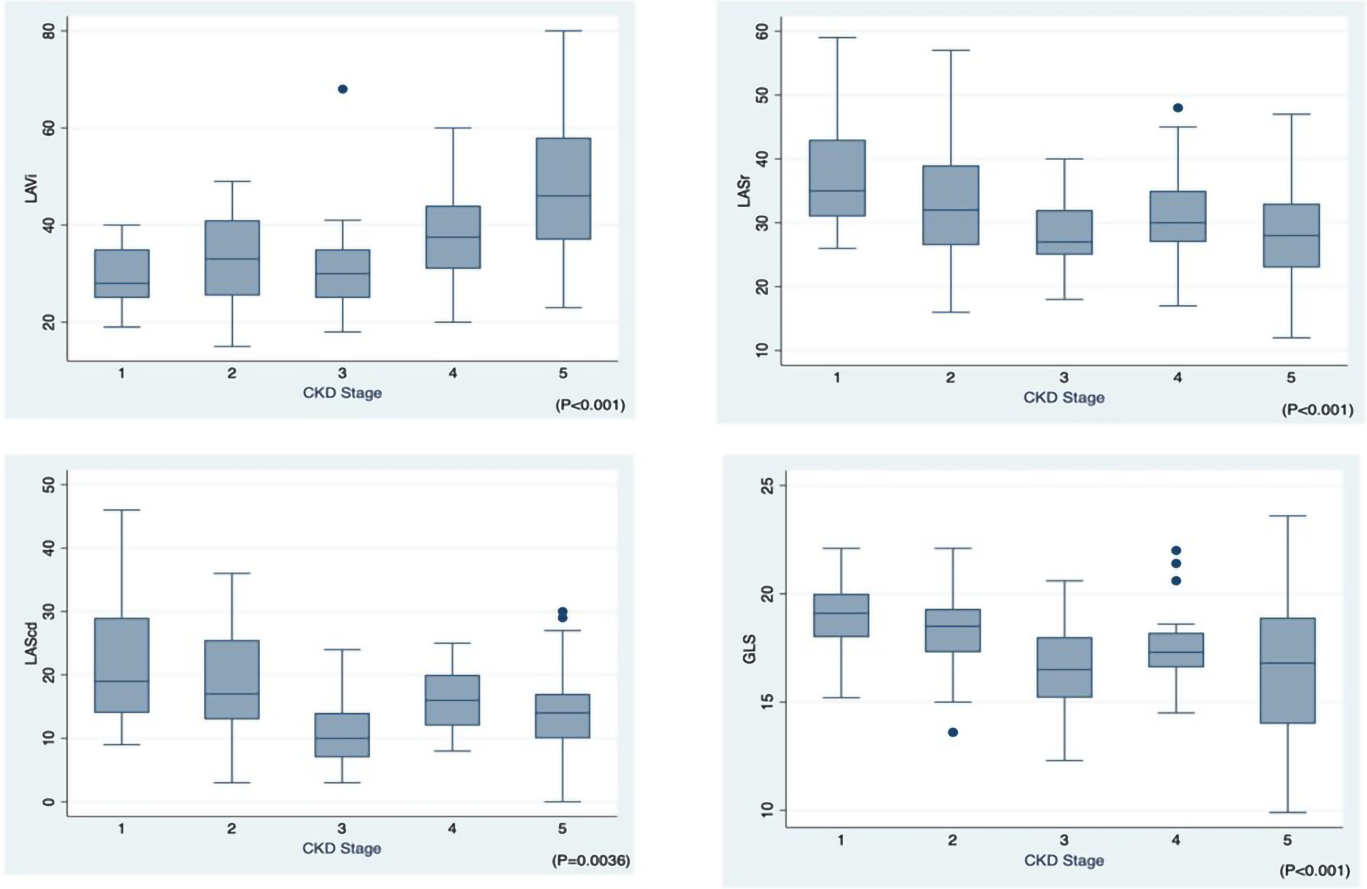
Left atrial volume index (LAVi), left atrial phasic strains reservoir phase (LASr), left atrial phasic strains conduit phase (LAScd), global longitudinal strain (GLS) across CKD stages 1–5.

Figure 2 shows the progressive reduction of LV and LA strain and increase in LA volume according to the CKD stages. The analysis of echocardiographic findings in Table 3 shows that in CKD patients, the stage of renal failure was associated with the increase in LVDs, LVDd, LV mass, E/e' ratio, TR max, LVEDV and LVESV (*p* < 0.05). The differences in GLS (%) (*p* < 0.001) among different CKD groups were also observed.

**Table 3:** Left ventricular volume and function across the CKD stages.

	Stage 1 (*n* = 21)	Stage 2 (*n* = 28)	Stage 3 (*n* = 27)	Stage 4 (*n* = 22)	Stage 5 (*n* = 71)	*p*-value
LVDd, mm, mean (SD)	45.7 (3.2)	45.4 (3.0)	43.5 (4.9)	47.7 (3.6)	48.7 (5.1)	**<0.001**
LVDs, mm, mean (SD)	29.0 (2.8)	29.6 (2.9)	27.6 (5.1)	30.5 (2.7)	32.3 (4.9)	**<0.001**
LVEF, %, median (IQR)	64 (63–67)	65 (62.5–67)	63 (59–65)	65 (62–66)	63 (60–66)	**0.0210**
LVEDV, ml mean (SD)	66.2 (15.1)	71.4 (12.9)	62.4 (17.7)	80.7 (22.2)	91.9 (30.6)	**<0.001**
LVESV, ml mean (SD)	21.8 (6.5)	25.5 (5.8)	23.1 (8.0)	28.45 (9.6)	35.4 (14.8)	**<0.001**
LV mass, g/m^2^, median (IQR)	89 (76–114)	91 (82–103.5)	93 (80–123)	107 (88–146)	154 (121–190)	**<0.001**
GLS, %, mean (SD)	−19.0 (1.7)	−18.2 (2.1)	−16.6 (1.9)	−17.7 (1.8)	−16.5 (2.9)	**<0.001**
E, cm/s, median (IQR)	78 (60–94)	72 (54–93)	60 (52–72)	71.5 (62–83)	78 (65–96)	**0.0257**
A, cm/s, median (IQR)	69 (62–83)	70.5 (58.5–95.5)	81 (74–87)	84.5 (75–104)	90 (76–113)	**<0.001**
e’, cm/s, mean (SD)	11.6 (3.6)	10.2 (3.3)	8.2 (1.9)	9.1 (1.8)	8.2 (2.3)	**<0.001**
E/e’, mean (SD)	6.8 (1.6)	7.3 (3.1)	7.9 (2.3)	8.2 (3.1)	10.7 (4.3)	**<0.001**
IVRT, ms, median (IQR)	110 (103–120)	111 (101–122)	119.2 (108–126)	110 (104–120)	107 (93–116)	0.0630
TR max, cm/s, mean (SD)	2.3 (0.2)	2.3 (0.2)	2.3 (0.3)	2.5 (0.3)	2.6 (0.3)	**<0.001**
s’, cm/s, mean (SD)	9.1 (0.7)	9.3 (0.9)	9.3 (1.0)	9.9 (1.3)	10.4 (1.3)	**<0.001**
PAPs, mmHg, mean (SD)	29.6 (3.3)	31.0 (4.6)	31.1 (4.1)	34.6 (6.8)	36.7 (6.2)	**<0.001**

Values are mean (SD), *n* (%), or median (interquartile range). The Jonckheere Terpstra test was used to assess for trend in continuous variables across the CKD stages. The chi-square test was used to assess the difference in categorical variables across the CKD stages. A *p* value <0.05 (bold) was considered to be statistically significant.

The analysis of LA volume and strain in Table 4 shows that higher severity of CKD stages was associated with higher values of LAd (mm) (*p* < 0.001), LA strain in four-chamber view (LAS 4C; %) (*p* < 0.001), LA strain in two-chamber view (LAS 2C; %) (*p* < 0.001), LA volume in four-chamber view (LAS 4C; %) (*p* < 0.001), LA volume in two-chamber view (LAV 2C; %) (*p* < 0.001), LA volume index (LAVi; ml/m^2^) (*p* < 0.001), LA volume before A wave (LAV preA; ml) (*p* < 0.001). The significant reduction in the LA reservoir strain (LASr; %) (*p* < 0.001), while the remarkable increase (less negative) was observed in the LA conduit strain (LAScd; %) (*p* < 0.001) among different CKD groups. The alteration of LASct across CKD stages was not significant (*p* = 0.2144).

**Table 4:** Left atrial volume and strain across the CKD stages.

	Stage 1 (*n* = 21)	Stage 2 (*n* = 28)	Stage 3 (*n* = 27)	Stage 4 (*n* = 22)	Stage 5 (*n* = 71)	*p*-value
LAd, mm, mean (SD)	33.6 (3.7)	34.6 (4.7)	33.8 (4.7)	35.7 (5.2)	39.3 (5.0)	**<0.001**
LAS 4C, %, median (IQR)	15.2 (15.2–15.2)	17.6 (16.2–18.1)	14.3 (13.7–15.2)	20.1 (19.9–20.2)	21.3 (18.0–24.2)	**<0.001**
LAS 2C, %, median (IQR)	14.0 (14.0–14.0)	15.7 (14.3–16.5)	14.2 (13.4–15.9)	17.9 (17.5–18.2)	19.9 (16.7–22.5)	**<0.001**
LAV 4C, %, mean (SD)	41.3 (6.1)	48.4 (12.4)	42.6 (17.3)	62.5 (11.3)	70.9 (23.8)	**<0.001**
LAV 2C, %, mean (SD)	41.1 (6.9)	44.9 (10.7)	40.0 (11.8)	57.3 (11.4)	63.9 (18.4)	**<0.001**
LAVi, ml/m^2^, mean (SD)	29.1 (6.0)	33.25 (9.5)	30.9 (9.3)	38.3 (10.3)	48.1 (13.1)	**<0.001**
LASr, %, mean (SD)	37.4 (8.3)	33.5 (10.0)	27.6 (5.6)	31.4 (7.2)	28.1 (7.9)	**<0.001**
LAScd, %, mean (SD)	−22 (9.8)	−18.6 (8.5)	−10.9 (5.3)	−15.8 (4.8)	−13.9 (6.7)	**<0.001**
LASct, %, mean (SD)	−15.5 (6.0)	−15.1 (5.1)	−16.5 (6.6)	−15.5 (4.5)	−14.3 (5.4)	0.2144

Values are mean (SD), *n* (%), or median (interquartile range). The Jonckheere Terpstra test was used to assess for trend in continuous variables across the CKD stages. The chi-square test was used to assess the difference in categorical variables across the CKD stages. A *p* value <0.05 (bold) was considered to be statistically significant.

Table 5 shows the results of multivariate regression models. Among echocardiographic parameters, LVEF (*β *= 0.068; *p* = 0.022), LV mass (*β* = 0.112; *p* = 0.001) and LASr (*β* = −0.077; *p* = 0.034) were found to be significantly correlated with GFR reduction.

**Table 5:** Multivariate analysis to determine independent echocardiographic predictors of reduced GFR.

Predictors	Beta	*p*	Coefficient	Robust standard error
LVDd, mm	−0.091	0.065	−0.028	0.015
LVDs, mm	0.052	0.238	0.016	0.014
LVEF, %	0.068	**0.022**	0.020	0.008
LV mass, g/m^2^	0.112	**0.001**	0.003	0.009
GLS, %	−0.007	0.804	−0.004	0.017
E, cm/s	−0.039	0.093	−0.002	0.0014
E/e’	0.035	0.071	0.107	0.059
TR max, cm/s	0.022	0.395	0.106	0.124
LAd, mm	0.084	0.129	0.0284	0.0185
LAVi, ml/m^2^	−0.006	0.879	−0.007	0.004
LASr, %	−0.077	**0.034**	−0.021	0.009
LAScd, %	0.011	0.770	0.002	0.007
LASct, %	−0.009	0.825	−0.003	0.016

LVDd, left ventricular diastolic diameter; LVDs, left ventricular systolic diameter; LVEF, left ventricular ejection fraction; LV, left ventricle; GLS, global longitudinal strain; E, e wave velocity; TR max, tricuspid regurgitation maximal velocity, cm/s; LAd, left atrial diameter; LAVi, left atrial volume index; LASr, left atrial strain reservoir; LAScd, left atrial strain conduit; LASct, left atrial strain contraction. A *p* value <0.05 (bold) was considered to be statistically significant.

## 4. Discussion

This study contributed to the current body of literature about the progressive reduction of LV and LA strain and increase in volume according to the CKD stages. By speckle tracking echocardiography, the current study also found the associations between left atrial reservoir function assessed (LASr), left ventricular ejection fraction (LVEF) and left ventricular mass index (LV mass) and the decline of kidney filtration function (GFR).

Left ventricular hypertrophy, dilatation, and dysfunction are the most common cardiac abnormalities in CKD patients (18). Previous studies found that left ventricular hypertrophy was the first prominent cardiac impairment due to a consistently high level of plasma urea (19), which is progressively more severe across CDK stages (20), earlier than dilatation and dysfunction (6, 18). In our study, GLS increased with the severity of CKD. This phenomenon could be explained that LV strain was sensitive to the preload and fluid overload condition and uremic cardiomyopathy (21).

The LV pathological changes may cause LA function impairment as there is a close connection between the two chambers. The left atrium is a more sensitive chamber to fluid overload and increased LV filling pressure among CKD patients (4). Left atrial volume index (LAVi) is an important predictor of cardiovascular and heart failure outcomes (22, 23) and left atrial strain also plays a novel maker in CKD (24). During the cardiac cycle, the left atrium plays as a reservoir to accumulate blood from pulmonary veins. After the mitral valve opens starting the diastolic phase, it serves as a conduit to let blood from LA to LV and the remaining blood in the LA will be pushed to LV by LA contractile force (25). In our speckle tracking echocardiography study, LA reservoir (LASr) and LA conduit (LAScd) function declined in parallel with kidney function, however, the LA pump function (LASct) did not follow this trend. In addition, this study found that among left atrial speckle tracking parameters, LA reservoir conduit functions was an important indicator that was associated with the severity of CKD. Therefore, it can be implied that these echocardiographic speckle-tracking parameters might be more sensitive to fluid overload at the early stages of CKD. This finding was similar to a previous report that worsening renal function was independently related to impaired LV and LA strain in CKD patients, and eGFR was associated with GLS, LASr and LAScd (26). However, in our study, we found that LVEF, and LV mass parameters but not LV GLS were predictors of GFR decline. In literature, GLS has been widely demonstrated its superior prognostic values in projecting cardiovascular adverse events or risk of mortality among CKD patients (27), even in comparison with LVEF (28). However, its role in predicting CKD progression is debatable. A recent study by Rupal Mehta et al. (2023) on 2,134 patients found that left ventricular longitudinal strain was not an independent predictor for a 30% reduction in eGFR over 7 years (29). Meanwhile, Ramesh Sankaran et al. indicated that hemodialysis patients were more likely to have impaired LVEF and GLS in comparison with those undergoing drug therapy (30). Indeed, the issue that patients having normal LVEF but abnormal GLS had been reported elsewhere (29, 31), which could be explained by the matter that the concept of longitudinal strain pertains to the assessment of contractile functionality at the tissue level along a specific axis, whereas LVEF expressed that alterations in left ventricular volumes resulting from myocardial contraction (32). In addition, our finding could be explained by the matter of small sample size and the homogeneity of our samples. This controversial issue should be elaborated on further research with larger sample sizes and in multiple centers. Hayer et al. (33) tried to understand the myocardial abnormalities across stages of CKD using the magnetic resonance imaging technique and they found that native myocardial T1 times (a biomarker of diffuse fibrosis) increased from stage 2–5, after being adjusted to hypertension and aortic distensibility. They also demonstrated that eGFR was a significant predictor of native myocardial fibrosis (33). Collectively, findings from our study suggest that LA strain parameters should be collected by speckle tracking echocardiography together with the measurement of LV mass and LV ejection function as it might indicate earlier pathophysiological changes which could be served as a maker to prevent or slow the progression of CKD.

Our study has several limitations. This study was unable to show changes in LV and LA over time due to its cross-sectional design. Secondly, the small sample size in this study hindered the ability to apply findings to the general population. In addition, the overwhelm of patients in CKD stage 5 might cause selection bias. Thirdly, although no difference in the selection of *P* wave as the time reference for defining zero-baseline for both LV and LA strain curves in comparison with the use of ventricular end-diastole, further research that compares both approaches should be elucidated. In addition, 2D-STE analysis has several intrinsic limitations, such as the dependency on the temporal stability of tracking patterns, the need for high-quality grey-scale images for reducing inter- and intra-observer variability of tracking data and finally a relevant intervendor variability. Finally, taking into consideration the fact that individuals with advanced stages of CKD demonstrated a higher likelihood of presenting with more severe hypertension, it is plausible that the efficacy of hypertension treatment could influence the outcomes of our study.

## 5. Conclusion

LV and LA volume and strain assessed by speckle tracking echocardiography were associated with advancing CKD stages. LV mass, LV ejection fraction and LA reservoir might be used to suggest the decline of kidney glomerular filtration function. They could be used at the earlier stages of CKD patients for better follow-up of GFR decline.

## Data Availability

The raw data supporting the conclusions of this article will be made available by the authors, without undue reservation.
